# Urinary equol concentration, body composition, and lifestyle factors were associated with bone mass in young women

**DOI:** 10.1186/s12905-025-04098-z

**Published:** 2025-12-04

**Authors:** Hiromi Hanano, Takumi Aoki, Shota Sasaki, Hiroyoshi Fujikawa, Kan Oishi, Yuiko Yamamoto, Hiroki Yamaguchi, Takaaki Mori, Kojiro Ishii

**Affiliations:** 1 Graduate School of Health and Sports Science, Doshisha University, Kyotanabe, Japan; 2 Faculty of Education, Miyagi Gakuin Women’s University, Sendai, Japan; 3 Faculty of Human Science, Hokkaido Bunkyo University, Sapporo, Japan; 4 Faculty of Human Health, Sonoda University, Amagasaki, Japan; 5 Faculty of Health and Sports Science, Doshisha University, Kyotanabe, Japan; 6 Faculty of Education, Saga University, Saga, Japan

**Keywords:** Women’s health, Osteoporosis, Prevention, University students.

## Abstract

**Background:**

Osteoporosis, a disease characterized by decreased bone strength and increased risk of fracture, represents a significant health concern in older adults. Primary osteoporosis prevention requires increasing bone mass (BM) to its peak at a young age. The estrogen-like effect of equol has been reported to suppress bone loss in postmenopausal women. This study aimed to investigate the relationship between BM and the urinary equol concentration, body mass, skeletal muscle mass, exercise habits, and menstrual abnormalities in younger women.

**Methods:**

Of a total of 395 female university students recruited, the final analysis included 275 participants after excluding those taking hormonal agents, those who did not undergo an equol test, and those who did not respond to the questionnaire. The mean age of the 275 participants was 20.1 years (± 1.1). BM was measured in the right calcaneus using an ultrasonic bone densitometer. BM was assessed using the osteo-sonoassessment index of the right calcaneus. Body composition was measured via the bioelectrical impedance method, using a multifrequency body composition analyzer. Hormone use, menstrual cycle, current exercise habits, and daily soy intake were assessed using self-administered questionnaires. Urinary equol concentration was measured using a Soy-Check system. Multiple regression analysis, using the forced-entry method, was performed with BM as the objective variable; and age, body mass index, skeletal muscle mass index, soy intake, exercise habits, menstrual cycle, and urinary equol concentration as the explanatory variables.

**Results:**

In our multiple regression analysis with osteo-sonoassessment index as the objective variable, the significantly associated factors were determined to be urinary equol concentration (β = 0.11, *p* < 0.05), skeletal muscle mass index (β = 0.29, *p* < 0.01), and current exercise habits (β = 0.31, *p* < 0.01). By contrast, age, body mass index, soy intake, and menstrual cycle were not found to be significantly associated with osteo-sonoassessment index (*p* > 0.05).

**Conclusions:**

Young women with higher urinary equol concentration, exercise habits, and skeletal muscle mass index had higher levels of BM. The acquisition of maximal BM at a young age is protective against osteoporosis; therefore, increased urinary equol concentration at a young age may be associated with better bone health and could potentially contribute to osteoporosis prevention.

## Background

 Osteoporosis, a disease characterized by decreased bone strength and an increased risk of fracture [1], is an important health problem in older adults. Falls and fractures account for 13.9% of cases requiring nursing care in Japan [2], and can significantly impact quality of life (QOL) [3]. In particular, women are at a higher risk of fractures following menopause when estrogen secretion declines [4], causing bone loss. Consequently, preventive measures against osteoporosis are important for women. The timing of peak bone mass (BM) attainment may vary based on the bone site [5]. However, a review of bone acquisition during infancy, childhood, and adolescence indicated that the peak BM is typically attained around the age of 20 [6]. Therefore, to prevent osteoporosis, it is important to increase the maximum BM in adolescence and minimize bone loss later in life [7, 8]. Lifestyle factors, such as nutrition and exercise during adolescence, have a significant impact on the acquisition of sufficient maximum BM. In addition, as age-related loss of muscle mass also increases the risk of falls and fractures [9], increasing muscle mass at a young age can help prevent falls and fractures in old age as well.

Equol is a compound structurally similar to estrogen and is produced as a metabolite of daidzein [10, 11], a soy isoflavone, through the metabolic activity of specific gut bacteria [12, 13]. Equol exhibits both estrogenic and anti-estrogenic effects. Due to its estrogen-like activity, anti-estrogenic effects, and potent antioxidant properties, equol is considered an important protective agent against symptoms related to aging in women, such as menopausal disorders, BM reduction, metabolic diseases, and lifestyle-related diseases. However, the amount of equol produced varies among individuals, and is influenced by genetic factors and the composition of the gut microbiota [14]. Research on isoflavone pharmacokinetics shows significant individual variability in metabolism, with not all adults being “equol producers” and capable of converting daidzein into equol [12, 15, 16]. Recent studies have suggested a decrease in the amount of equol produced among younger generations in Japan, which may be attributed to changes in the dietary habits [17]. The reduction in equol production may result in diminished health benefits, leading to more severe menopausal symptoms and an increased risk of osteoporosis and cardiovascular diseases in postmenopausal women.

Equol has been shown to be effective at preventing bone loss in postmenopausal women. Following one year of soy food consumption by postmenopausal women, one study found that the BM of the women who were capable of producing equol was less reduced than that of the non-producers [18]. Equol has properties that are similar to estrogen—a female hormone [10, 11]. Decreased estrogen levels are believed to affect bone and calcium metabolism, leading to bone loss and osteoporosis [19]. Therefore, equol, with its female hormone-like effects, may be effective for maintaining and increasing BM. However, the relationship between BM and the ability of young women to produce equol at the time of maximal BM remains unclear. Furthermore, nutrition, exercise, menstruation, and other factors may affect maximal BM, and should therefore be included in any studies regarding the effect of equol on BM. This study aimed to investigate the relationship between BM and the urinary equol concentration, body mass, skeletal muscle mass, exercise habits, and menstrual abnormalities in young women.

## Methods

### Participants

**Fig. 1:**
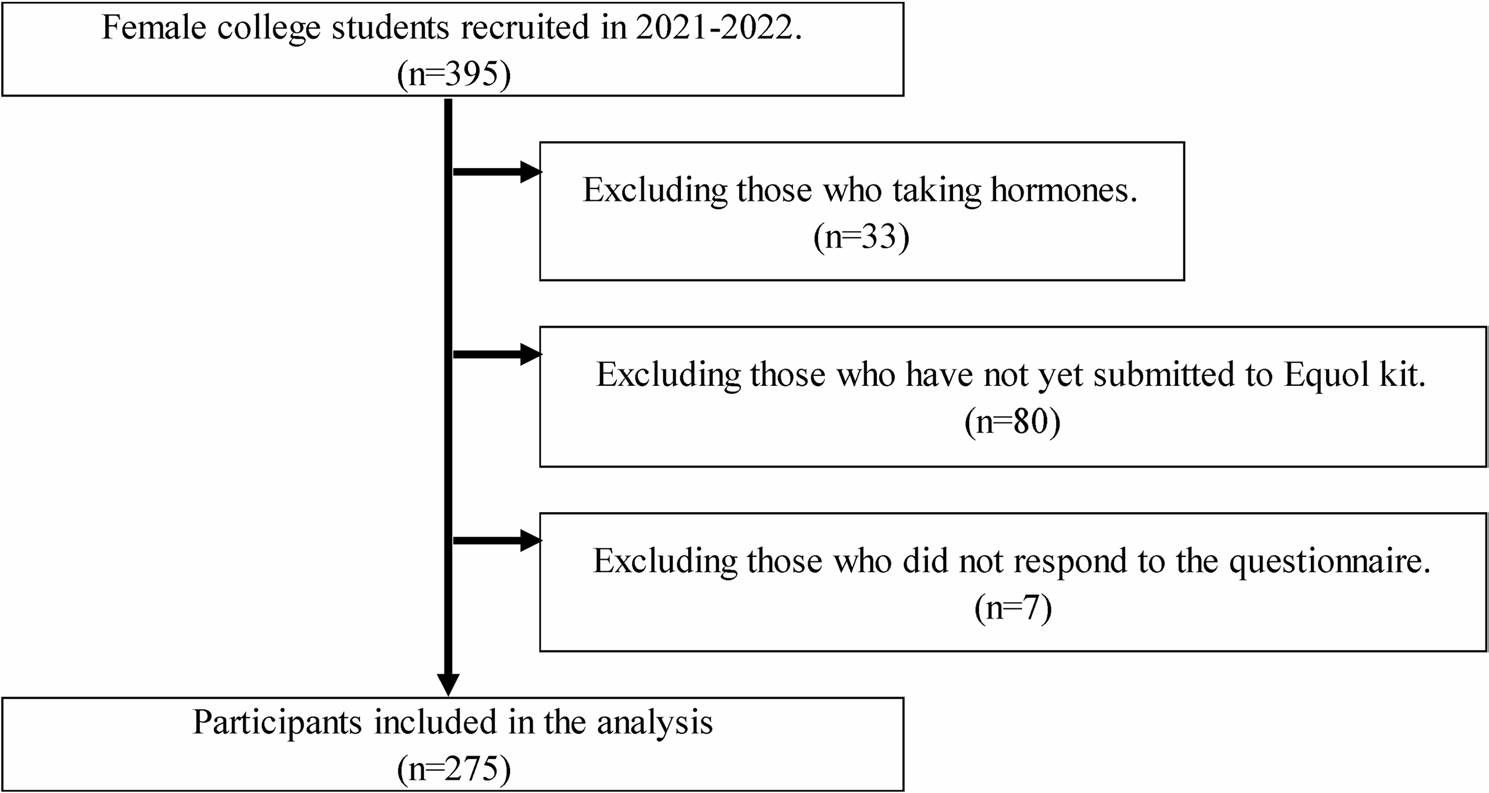
Participant selection

Between 2021 and 2022, 395 female university students aged 18–25 years from Doshisha University, Sonoda Women’s University, and Miyagi Gakuin Women’s University were surveyed using measurements and questionnaires. The purpose of this study was explained both orally and in writing, and written informed consent was obtained from all participants. The study was approved by the Doshisha University Human Subjects Research Ethics Committee (approval number: 20049) and conducted in accordance with the Declaration of Helsinki. The final analysis included 275 participants—excluding those taking hormonal agents, those who did not undergo an equol test, and those who did not respond to the questionnaire (Fig. 1).

### BM measurement

BM was measured using the AOS-100SA ultrasound bone densitometer (FUJIFILM Co., Ltd., Tokyo, Japan), which is simple to operate and does not expose the patient to radiation exposure. The ultrasonic bone densitometer has been shown to correlate with the gold standard dual-energy X-ray absorptiometry (DXA) method for BM measurement and its usefulness has been confirmed [20, 21]. The osteo-sonoassessment index (OSI) of the right calcaneus was used as the main evaluation item. It was calculated from the speed of sound (SOS) and transmission index (TI) of the bone, using the equation: OSI = TI × SOS [2]. A higher SOS indicates higher bone density and a higher TI indicates higher BM [22]. Since the right calcaneus is known to correlate with the total body BM [23], BM was measured once on the right calcaneus in this study. BM was assessed using OSI of the right calcaneus.

### Measurement of the urinary equol concentration

Participants were given the “Soy Check” [24–26] equol test kit (Healthcare Systems Co., Ltd., Aichi, Japan). All of the participants consumed soy foods equivalent to approximately 100 mg of isoflavone between dinner and bedtime the day before the measurement and collected their first urine sample the following morning at home, using a tube from the kit [27]. Specifically, the participants recruited in 2021 consumed two 125 mL bottles of the soy beverage “Sugoi Daizu Original” (Otuka Food Co., Ltd., Osaka, Japan), and the participants recruited in 2022 consumed the soy supplement “Nature Made Soy Isoflavone” (Otuka Food Co., Ltd.). Following urine sample collection, each participant sent their test kit to a contracted laboratory (Health Care Systems Co., Ltd.) for analysis. Urinary equol concentration was calculated using creatinine-corrected values (µmol/g cre).

### Questionnaire

The participants self-reported their date of birth, current exercise habits, menstrual cycle, soy intake, and the use of hormone medication. Current exercise habits were evaluated by asking the participants whether they belonged to an athletic club. The normal range for menstrual cycles was considered to be 25–38 days [28–30]. Soy intake was calculated using the Food Frequency Questionnaire Based on Food Groups (FFQ [ver. 6]) (KENPAKUSHA Co., Ltd., Tokyo, Japan). The FFQ can be used to estimate nutrient intake for each food group, based on standard intakes and number of intakes per meal [31]. Its validity as a method for estimating nutrient intake has been confirmed previously [32].

### Body measurements and body composition

Height, weight, and appendicular skeletal muscle mass (ASM) were also measured. Body mass index (BMI) and skeletal muscle mass index (SMI) were measured via bioelectrical impedance analysis, using a Tanita MC-780 A body composition analyzer (Tanita Co., Ltd., Tokyo, Japan). SMI was calculated from the ASM and height, while BMI was calculated from the height and weight. ASM, height, and weight were measured using bioelectrical impedance analysis, and the values for SMI [33] and BMI were subsequently calculated based on these measurements. Since urine accumulation in the bladder can affect the body’s water content and potentially lead to inaccurate measurement results, participants were instructed to void before the measurement.

### Statistical analysis

Univariate regression analysis was performed using OSI as the objective variable and urinary equol concentration as the explanatory variable. Multiple regression analysis using the forced-entry method was also performed to examine factors related to BM—using urinary equol concentration, current exercise habits, normal menstruation, soy intake, BMI, and SMI as explanatory variables. According to Green [34], the minimum recommended sample size for multiple regression is *N* ≥ 50 + 8 m (where m is the number of predictors). With seven predictor variables, at least 106 participants are required. Our study included 275 participants, which exceeds this threshold and ensures sufficient statistical power. In addition, based on the commonly used rule of thumb requiring 10–15 participants per predictor variable, the minimum required number would be 70–105 participants, which is also satisfied in our study.

The assumptions of multiple regression were assessed using SPSS. Linearity and homoscedasticity were confirmed through the scatterplot of standardized residuals versus predicted values. Multicollinearity was evaluated using tolerance and the variance inflation factor (VIF), with all VIF values below 3.0. Independence of residuals was supported by the Durbin-Watson statistic (1.73), which falls within the acceptable range. Normality of residuals was verified by examining both the histogram and the normal probability (P–P) plot. All analyses were performed using IBM SPSS Statistics version 29.0 (IBM Japan Co., Ltd., Tokyo, Japan), and statistical significance level was set at *p* < 0.05.

## Results

Table 1 lists the characteristics of the 275 participants. The mean of OSI was 3.14 ± 0.42.

**Table 1:** Characteristics of study participants (*n*=275)

OSI	3.14	±	0.42	
Age (years)	20.1	±	1.1	
BMI (㎏/m^2^)	21.00	±	2.62	
SMI (㎏/m^2^)	7.10	±	0.83	
Soy intake (g/day)	50.47	±	40.41	
Urinary equol concentration (µmol/g cre)	1.66	±	4.67	
Exercise habit (Yes/No)	118 (43%)	/	157 (57%)	
Menstrual cycle (normal/abnormal)	206 (74%)	/	71 (26%)	

*OSI* Osteo-Sonoassessment Index, *BMI* body mass index, *SMI* muscle mass index, *SD* standard deviation

Mean ± SD, or number of persons (%)

Table 2 shows the results of the univariate and multiple regression analyses with OSI as the objective variable. In the univariate analysis, urinary equol concentration had a significant positive relationship with OSI (B = 0.01, *p* < 0.05; model a). In the multiple regression analysis with OSI as the objective variable, the significant associated factors were urinary equol concentration (β = 0.11, *p* < 0.05), SMI (β = 0.29, *p* < 0.01), and current exercise habits (β = 0.31, *p* < 0.01; model b). By contrast, age, BMI, soy intake, and menstrual cycle were not found to be significantly associated with OSI.

**Table 2:** Univariate and multiple regression analysis with OSI as the objective variable

	model a		model b		
	B	SE	B	SE	β
Urinary equol concentration (µmol/g cre)	0.01*	0.01	0.01	0.01	0.11*
Age (years)			–0.02	0.02	–0.06
BMI (㎏/m^2^)			0.01	0.01	0.05
SMI (㎏/m^2^)			0.14	0.04	0.29**
Soy intake (g/day)			0.00	0.00	–0.03
Exercise habit (Yes/No)			0.26	0.07	0.31**
Menstrual cycle (normal/abnormal)			–0.02	0.05	–0.02

*p＜0.05. ** *p*＜0.01. R^2^＝0.322 (model b). F＝18.139** (model b)

Model a indicates univariate regression analysis. Model b indicates multiple regression analysis

B: partial regression coefficient. SE: standard error. β: standardized partial regression coefficient

## Discussion

This study aimed to investigate the relationship between BM, body mass, skeletal muscle mass, exercise habits, menstrual abnormalities, and the ability of young women to produce equol. Higher SMI and exercise habits, as well as higher levels of urinary equol concentration, were found to be associated with higher BM in young women.

It has been previously reported that bone resorption was suppressed and bone loss was reduced in a cohort of postmenopausal women who were unable to produce equol when given an equol supplement [35]. Equol production in this study was also significantly associated with BM, although exercise habits and SMI were entered together into our multiple regression analysis. SMI and exercise habits are necessary for maximizing BM, and the ability to produce equol at a young age may help prevent osteoporosis and the need for assistance in older ages.

On the other hand, there are individual differences in equol production [14], and a comparison of the percentages of equol producers in different countries indicated that the percentage of equol production is higher in Asian populations than in Western ones [36]. However, in recent years, equol production in young Japanese women has declined compared to that in middle-aged and elderly women [37]. This decline has been attributed to a decrease in soy food intake, owing to a general Westernization of the Japanese diet [38]. However, it has also been reported that equol production may also be related to factors other than soy food intake [39, 40]. The reasons for the decreased level of equol production observed recently in young Japanese women therefore remain unclear. Our results from this study indicate that BM in youth is related to urinary equol concentration. In our analysis, β coefficients for SMI and exercise habits were higher than the coefficient for urinary equol concentration, highlighting the particularly strong contribution of these two factors to BM. Therefore, it appears that even women with low urinary equol concentration, such as those observed among young women in recent years, can compensate for the equol-related benefits to BM if they exercise regularly and have high SMI.

The OSI of the participants in this study was higher than the OSI reference value [41] (2.71 for the same age group, 20 years) with a range of 2 standard deviations (2SD) from 2.18 to 3.23. Exercise habits and muscle mass influence increases in BM. Bones have sensors that detect mechanical stresses such as strain. Exercise and other factors have been shown to increase BM when the bone strain exceeds a certain threshold. This is known as the mechanostat theory [42]. The present study also suggests that the association between exercise habits and BM is due to the mechanostat theory. Increases in BM involve not only mechanical stimuli during exercise, but also a number of other interactions between muscle and bone tissues [43]. Muscle and bone interaction means that when muscle mass increases or decreases, BM increases or decreases as well. A positive correlation has been reported between muscle mass and BM in a number of previous studies [44, 45]. Moreover, an increase in muscle mass stimulates an increase in BM, even during the developmental period [46]. Our results were also influenced by the interaction between muscle and bone, with higher SMI values indicating higher BM.

In this study, 26% of the participants had abnormal menstrual cycles. In a previous study conducted in Japanese female university students (aged 20.4 ± 0.9 years), 22% of the participants were found to have menstrual cycles beyond the normal range (25–38 days) [47]. Therefore, the results of this study are consistent with those of previous research and are not considered to be atypical. Menstrual cycles were included as an explanatory variable because previous studies have suggested that irregular menstrual cycles are associated with lower BM in young women [48]. However, in the present study, menstrual cycle status was not significantly associated with BM.

Nevertheless, this study had several key limitations worth noting. It was cross-sectional in design, and therefore not investigate effects and causal relationships. Second, the questionnaire was based on self-reporting; therefore, our results were susceptible to recall bias. Third, the participation rate for equol measurements may have decreased. The participants in this study were required to consume the equivalent of 100 mg of soy foods for the equol measurement, before collecting their first urine samples the following morning, which may have represented a cumbersome task for university students. Fourth, this study did not collect information on other potential confounding factors that may influence BM, such as overall dietary intake and smoking status, making it difficult to account for their effects.

However, we were able to clarify the relationship between BM and urinary equol concentration at a young age, which is useful for the prevention of osteoporosis and the need for additional assistance in older ages. In a previous study that investigated equol production capacity, participants in their 20 s were recruited at a gynecology clinic []. One of the major advantages of this study was that we recruited healthy female university students from a non-medical institution.

## Conclusion

Young women with higher urinary equol concentration, exercise habits, and SMI had higher BM. Achieving maximal BM at a young age is protective against osteoporosis; therefore, increased urinary equol concentration at a young age may be associated with better bone health and could potentially contribute to osteoporosis prevention.

## Data Availability

The datasets used and/or analyzed during the current study available from the corresponding author on reasonable request.
